# Dignity Therapy Training for the Healthcare Professionals: Lessons Learned From an Italian Experience

**DOI:** 10.3389/fpsyg.2022.859775

**Published:** 2022-07-25

**Authors:** Loredana Buonaccorso, Sara Alquati, Luca Ghirotto, Alice Annini, Silvia Tanzi

**Affiliations:** ^1^Scientific Directorate, Azienda USL—IRCSS di Reggio Emilia, Reggio Emilia, Italy; ^2^Palliative Care Unit, Azienda USL—IRCSS di Reggio Emilia, Reggio Emilia, Italy; ^3^Qualitative Research Unit, Scientific Directorate, Azienda USL—IRCCS di Reggio Emilia, Reggio Emilia, Italy

**Keywords:** dignity therapy, palliative care, physician patient-communication, nurse–patient relationship, hospital

## Abstract

**Introduction:**

Dignity therapy (DT) is brief psychotherapy targeting psychological and existential suffering among patients with a life-limiting illness. Studies have been conducted on the use of DT by healthcare professionals. In Italy, the current legislation defines that any form of psychotherapy may be performed exclusively by psychotherapists. Consequently, this intervention is unlikely to be used by other healthcare professionals. Herein, we will describe a training on DT not as a psychotherapy intervention but as a narrative intervention for non-psychotherapists health care professionals. Finally, we will explore the potential enablers/barriers as experienced by palliative care physicians and nurses.

**Methods:**

The study was conducted in the Psycho-Oncology Unit within the Cancer Research Hospital of Reggio Emilia (Italy). It consisted of an exploratory qualitative case study. Data were collected employing observations and interview data and thematically analyzed.

**Results:**

The training was attended by six physicians and ten nurses and took place during two-afternoon sessions for 10 h. Two participants put their training into practice and administered DT under the supervision of a psychotherapist. Data analysis highlighted five overarching themes relating to the training experience and direct use of DT, namely, (i) time required, (ii) psychological skills, (iii) patient’s disease awareness, (iv) patient’s life history, and (v) distinguishing DT from Advance Care Planning.

**Conclusion:**

Palliative care professionals found DT to be a valuable non-pharmacological hospital-based intervention to address the person beyond the patient and his clinical conditions. In our experience, considering that in Italy, psychotherapy is an intervention that psychotherapists can only perform, it can help organize different training on DT for psychotherapists and other healthcare professionals.

## Introduction

Palliative care aims to enhance the quality of life and reduce the suffering of patients and their families in the setting of terminal disease and advanced care (Hui and Bruera, 2018; Osman et al., 2018; World Health Organization, 2019). Beyond treating physical conditions (Sokol et al., 2020), care should be provided to address the patients’ psychological symptoms. Indeed, growing evidence has highlighted the need to address psychospiritual aspects such as distress, perceived loss of dignity, and perception of being a burden to family members (Sokol et al., 2020; Bluck et al., 2021). In this setting, dignity therapy (DT) is brief psychotherapy developed in response to evidence on the prevalence of psychological and existential factors such as hopelessness, the burden to others, and dignity in palliative care patients (Chochinov et al., 2005, 2011; Martínez et al., 2017). The therapy foresees an intensive format, where the healthcare professionals interact with the patients to collect their direct narration on their lives and the most significant moments or important thoughts they wish to preserve by creating a written Generativity Document (Chochinov, 2012). Therapy sessions are transcribed and edited, and the resulting Generativity Document is returned to patients, who can share it with their family, friends, and healthcare professionals. This intervention protocol features nine standard questions as prompts to invite the patients to begin their recollections about what they want to say. The questions help guide a conversation with a DT-trained healthcare professional (Chochinov, 2012; Table 1).

**TABLE 1:** Dignity therapy question protocol.

1	Tell me a little about your life history, particularly the parts that you either remember most or think are the most important. When did you feel most alive?
2	Are there specific things that you would want your family to know about you, and are there particular things you would want them to remember?
3	What are the most important roles you have played in life (family roles, vocational roles, community service roles, etc.)? Why were they so important to you, and what do you think you accomplished in those roles?
4	What are your most important accomplishments, and what do you feel most proud of?
5	Are there particular things that you feel still need to be said to your loved ones, or things that you would want to take the time to say once again?
6	What are your hopes and dreams for your loved ones?
7	What have you learned about life that you would want to pass along to others? What advice or words of guidance would you wish to pass along to your son, daughter, husband, wife, or parents, etc.?
8	Are there words or, perhaps, instructions you would like to offer your family to help prepare them for the future?
9	In creating this permanent record, are there other things that you would like included?

Once the taped session is completed, the patient’s recorded dialogue is reshaped into a narrative (Chochinov, 2012). The interview is first transcribed verbatim within the next 2–3 days. This transcript then undergoes formatting and editing, especially (1) essential clarifications (eliminating colloquialisms, non-starters, and portions of the transcript not related to generativity material); (2) chronological corrections; and (3) tagging and editing any content that might inflict significant harm or suffering on the transcript’s recipient(s) to produce a manuscript that patients feel captured their intent and achieved the appropriate final tone. Finally, once the edited transcript is completed, a second session is arranged for the therapist to read the entire document to the patient. This therapeutic session lasts between 30 and 60 min and is offered either at the patient’s bedside for those in hospital, or the case of outpatients, at the patient’s home, residence, or the therapist’s office. This step is often emotionally evocative for the patient as he/she hears their words, thoughts, and feelings spoken aloud. The patient is invited to make any editing suggestions, including identifying errors of omissions or commission.

To date, many studies have been conducted on the use of DT by psychologists, psychiatrists, nurses, social workers, and chaplains (Chochinov et al., 2005, 2012; Montross et al., 2011; Centek et al., 2018; Mai et al., 2018; Sokol et al., 2020; Korman et al., 2021; Schoppee et al., 2022) less frequently by palliative care physicians. A randomized controlled trial was conducted by Juliao, as a palliative care physician on the efficacy of DT on depression and anxiety in 80 terminally ill Portuguese patients (74 were patients with cancer). The data showed a beneficial effect on depression and anxiety symptoms in end-of-life care for patients assigned to the DT group (*N* = 29) over 30 days (Juliao et al., 2014).

One study occurred in the academic hospital setting as an initiative where DT was used with 12 patients at the end of life as an educational intervention for first-year psychiatry and family medicine residents completing a rotation in palliative care (Tait et al., 2010). According to the authors, DT provided a narrative that helps the patients make sense of their world amid approaching death (Tait et al., 2010).

Another study was conducted by pulmonologists with 10 patients with chronic obstructive pulmonary disease (COPD) at the end of life (Brozek et al., 2019), describing the utility of DT in recognizing and fulfilling the spiritual needs of patients.

Important reflections on the use of DT have also been reported by clinicians working with patients with dementia (Aspiras et al., 2019). The DT process offered an opportunity for a patient to feel honored and worthy despite his/her deteriorating physical condition. For the caregiver, it was an opportunity to shift from “this is not who my husband is” and become more comfortable with “this is who he is now.”

A recent randomized control trial was conducted on 144 advanced cancer patients at a private hospital in Kenya, showing that DT in addition to routine clinical care was not shown to statistically improve patients’ quality of life as compared to usual care only (Weru et al., 2020). In contrast, the data showed an improvement in the anxiety levels of the patients. The authors suggested that incorporating DT in clinical care, more so in palliative patients (especially those with anxiety) with advanced cancers, may be beneficial, but different counsellors approach subject matter differently. This could have been a major negative influence in the study since standardization of counseling technique is difficult (Weru et al., 2020).

Some studies have also been conducted in Italy by psychologists, psychotherapists (Iani et al., 2020; Buonaccorso et al., 2021), and nurses (Nunziante et al., 2021) on the use of DT both in palliative care and in other care settings.

A randomized, controlled trial was conducted with 64 terminally ill patients (33 were patients with cancer) who were randomly assigned to the intervention group (DT + standard palliative care) or the control group (standard palliative care alone) (Iani et al., 2020). This study provides initial evidence that patients in the DT intervention maintained similar levels peace of mind from pretest to follow-up (15–20 days after the baseline assessment).

A multicenter, retrospective, qualitative study was conducted to explore the meanings emerging from two DT questions (“*What have you learned about life that you would want to pass along to others?*”*;*“*Are there words or perhaps even instructions you would like to offer to your family to help prepare them for the future?*”), particularly salient to generativity, among patients with cancer in different care settings and stages of illness (Buonaccorso et al., 2021). Generativity is a process whereby patients nearing the end of life invest in those they will soon leave behind. Attending to generativity could encourage patients to reflect on the essential things in their lives to shape how they will be remembered (Chochinov et al., 2002). The following three themes and related meanings emerged from 37 DT sessions concerning the two questions: (1) meanings concerning the present life and illness, including the experience of suffering; (2) thoughts and actions toward the self, including ways in which the patients have felt alive; and (3) thoughts and actions toward significant others, especially values that are based mainly on love for oneself and others. No notable differences across stages and care settings emerged regarding the meanings emerging from the two DT questions. The data showed that conversations about generativity could inform clinicians on how to communicate existential and meaning-based issues across different stages of illness (Buonaccorso et al., 2021).

One study assessed the feasibility and acceptability of a nurse-led DT intervention in patients with advanced cancer who received palliative care in an Italian hospital (Nunziante et al., 2021). Twenty-eight patients completed the DT intervention. At the end of the study, no statistically significant reduction in dignity-related distress was observed; most of the patients found DT helpful and satisfactory (Nunziante et al., 2021).

Considering these promising results, we wanted to raise awareness of the use of DT in palliative care Italian services by implementing a training program for clinicians working in hospital and home care services. In Italy, the current legislation defines that any form of psychotherapy may be performed exclusively by psychotherapists (Ministero dell’istruzione dell’università e della ricerca [MIUR], 1989). Consequently, this intervention is unlikely to be used by other healthcare professionals.

Nonetheless, according to the definitions adopted by the European Association of Palliative Care (2011)^1^ and the World Health Organization (2019)^2^, addressing the patient’s psychosocial aspects and offering psychological support to patients and families are essential parts of palliative care. In contrast to medical and nursing care, provided by physicians and nurses, psychological support in palliative care is not exclusively assigned to psychologists. On the contrary, it is expected that all professionals working in palliative care acquire basic knowledge of the psychological dynamics at work in life-limiting disease and related skills in communication and psychological risk assessment (Jünger and Payne, 2011).

In this study, we did not invite non-psychotherapists to do therapy, but to use DT-inspired features for providing palliative care. Beside, herein we describe a DT’s training as a narrative, non psychotherapeutic intervention targeting healthcare professionals. Finally, we explore the potential enablers/barriers for using those intervention’s feature as experinced by physicians and nurses working in palliative care.

## Methods

### Design

This was an exploratory qualitative case study design (Braun and Clarke, 2006; Baxter and Jack, 2008; Walshe, 2011) using a triangulation of field notes and semi-structured interviews. This design explores those situations where the intervention to evaluate has no clear, single set of predicted outcomes.

### Case Definition

The case was defined as the intensive DT training program of the Psycho-Oncology Unit, supporting the palliative care specialists to improve their competency in using it. The program foresaw a post-training application experience of DT by trainees supervised by the psychologist psychotherapist (LB).

### Setting

The Psycho-Oncology Unit of the Local Health Authority, based within the Cancer Research Hospital of Reggio Emilia (Italy), is a specialized unit that includes six psycho-oncologists. It was established in 2016 for oncological inpatients and outpatients and their families, with the aim of (i) providing psychological intervention to patients and their families; (ii) organizing advanced training on psychosocial skills for healthcare professionals; and (iii) conducting research on psycho-oncology, palliative care, and bereavement.

### Participants

Eligible participants of this case study were physicians and nurses attending the training and working in hospital and home palliative care services.

### Data Collection

Data were collected by an external researcher’s (AA) observation of participant trainees and through semi-structured interviews with trainees inquiring about their use of the instrument, which lasted for 20 min (Table 2). Field notes were derived from observations of the: (i) training sessions; (ii) DT’s administration by trainees; and (iii) supervision meetings on DT’s administration.

**TABLE 2:** Interview guide.

1	Can you tell us about your experience with doing Dignity Therapy?
2	During your intervention using Dignity Therapy, which factors facilitated your work with the patient?
3	During your intervention using Dignity Therapy, which factors hindered your work with the patient?
4	How did you select the patients whom you assigned for Dignity Therapy? At which point/phase of the disease path did you decide to conduct Dignity Therapy?

### Data Analysis

The data were analyzed using thematic analysis (Braun and Clarke, 2006). Transcripts of interviews and observational field notes were analyzed by a psychotherapist (LB), and themes were iteratively discussed and refined (Baxter and Jack, 2008; Walshe, 2011), with the co-author (LG) having senior experience in qualitative research.

The researcher employed the following steps:

- focus (repeated reading) on the field notes taken during observation of the participant while applying DT with the patient;
- generation of open codes by identifying and labeling particular features and concepts within the cases that were considered relevant to the research’s aim;
- grouping of relevant codes into themes;
- review and discussion of themes with co-authors;
- refining and organizing themes were finally into a coherent narrative.

### Ethical Considerations

Because the study addressed educational practices and quality improvement, the hospital’s internal regulation of the competent body of the hospital (CME Scientific Committee) did not require specific informed consent procedures. Nevertheless, all participants’ information was handled as confidential, and informed participants’ consent was verbally gathered throughout. Trainees were also informed about confidentiality and reassured that the reporting of their observations would be carried out anonymously and that statements given would in no way affect them professionally.

## Results

### Training Experience

The training was organized as a CME workshop (15 CME credits) led by a specialist (LB) from the Psycho-Oncology Unit, who had specifically trained on DT at an international workshop held in Winnipeg, Canada (2011) and has been applying this brief psychotherapy since 2012 (Buonaccorso et al., 2021, 2022). The course, which took place during March and April 2019, was addressed to palliative care professionals and was attended voluntarily by 16 participants, among which six were physicians (five from the oncological hospital and one from home-based palliative care), and ten were nurses (two from the oncological hospital and eight from home-based palliative care).

The syllabus included theoretical lectures providing basic notions on the DT (Chochinov, 2012) and interactive discussions on its use and was spread out over two-afternoon in-class modules (5 h each). In line with the purpose of the course, the trainer encouraged participants to plan an intermediate step of supervised application of the instrument between the first- and second-course module during which participants could put into practice the newly acquired notions and further develop their practical skills. This intermediate step of supervised application was planned and discussed interactively by participants during the first meeting (each participant was invited to propose possible ways of applying DT on how he/she would have specifically applied DT) and again at the second meeting to share and provide feedback on their experience.

While most participants expressed interest in the supervised application, only two participants eventually adhered to the initiative. The two professionals were from the Specialized Palliative Care Team of the hospital and involved one cancer patient each. DT was intentionally performed separately from the clinical evaluation.

Before the end of the second training module, the trainees discussed the relevance of the skills acquired and their significance in their area of expertise and concerns about the possibility of application. Some of the concerns were relevant to the point they withheld participants from attempting its application during a tutored DT session.

In particular, we describe the data from the application of the DT process by two palliative care physicians and how other trainees were influenced and used what they had learned more straightforwardly.

### Thematic Analysis

From the overall analyses of the data collected, five themes emerged, namely, (i) time required; (ii) psychological skills; (iii) patient’s disease awareness; (iv) patient’s life history; and (v) distinguishing DT from Advance Care Planning. Below, we discuss each theme in terms of the barriers and enablers (Table 3).

**TABLE 3:** Summary of the results.

Themes	Barriers	Enablers
Time required	It is difficult to schedule a dedicated time for DT during clinical consultations, especially for administering the intervention according to the protocol (recording, transcription, editing). It is necessary more time to learn how to “handle” DT and its psychological implications.	It supported an empathetic approach, use some DT questions learned in the regular communication with patients. The time spent to during DT was viewed as enriching, since the communication was entirely dedicated to the patient’s life history and not to the clinical aspects.
Psychological skills	It is necessary to implement psychological skills before feeling comfortable in practicing DT.	Previous training on psychological skills and spirituality interventions (acquired over the years) as useful in administering DT. Emphasis on this aspect was associated to local resources available or lack thereof.
Patient’s disease awareness	The nine questions refer to legacies, so the practice of DT might entail the risk of potential distress, especially when the patients are not aware of their prognosis.	The patients’ awareness of their disease emerged as a facilitating factor to administer DT.
Patient’s life history	The lack of knowledge of possible critical events in the patient’s family entailed ‘risks’ for the trainees in proposing DT since eliciting the narrations can bring back to the surface especially emotional memories.	Investigating the family history before DT administration was an added value for healthcare professionals, as it allowed the professional to be prepared to the patient’s emotional states.
Distinguishing dignity therapy from advance care planning	This realization appeared to be particularly linked to the role of palliative care physicians who usually investigated patients’ will.	The concept of DT also as a useful intervention in understanding the patients’ values, providing the basis on which to plan subsequent Advance Care Planning.

*DT, Dignity Therapy.*

#### Time Required

##### Barriers

Time appeared to be one of the primary reasons withholding most of the participants from applying for DT. Trainees reported they could not schedule a dedicated time for DT during clinical consultations, especially for administering the instrument according to the protocol (recording, transcription, and editing). Most participants shared this comment, and each participant’s emphasis on this aspect depended on local resources available and the organization of services.

Finally, time was also mentioned in that the trainees perceived they needed more time to learn how to “handle” DT and its psychological implications.

##### Enablers

Several trainees reported that, aside from the DT setting, they had benefitted from the time in training also in their daily practice. They had introduced some of the DT questions learned in their regular communication with their patients, which they attributed an empathic approach to the doctor/nurse–patient relationship.

As to the time aspect described by the two palliative care physicians who had carried out DT with their patients, the time spent during DT was viewed as enriching since the communication was entirely dedicated to the patient’s life history and not to the clinical aspects. They suggested that having a medical trainee in charge of the interview transcription would be essential in optimizing the time doctors were engaged in DT.

#### Psychological Skills

##### Barriers

Many trainees felt their basic psychological skills were inadequate for administering DT and felt the need to implement those skills before feeling comfortable in practicing DT. Their statements suggested that having DT-trained psychotherapist or other operator on the team to supervise them as they administer DT would be an opportunity to implement this intervention. Again, emphasis on this aspect was associated with local resources available, or lack thereof. The need for a time was also mentioned about mastering the technique.

##### Enablers

The two palliative care physicians who administered DT evidenced their previous training in psychological skills and spirituality interventions (acquired over the years) as helpful in administering DT.

#### Patient’s Disease Awareness

##### Barriers

One of the barriers highlighted was the patient’s awareness of their prognosis. Because DT questions refer to legacies, that is, to the things that patients would like to leave to those close to them, the practice of DT might entail the risk of potential distress, especially when the patients are not aware of their prognosis. Trainees suggested that DT could only be used in patients who are aware of their prognosis.

##### Enablers

Conversely, the patients’ awareness of their disease emerged as a facilitating factor to administer DT.

#### Patient’s Life History

##### Barriers

The lack of knowledge on possible critical events in the patient’s family entailed a “risk” for the trainees since eliciting narrations can bring back to the surface incredibly emotional memories.

##### Enablers

Investigating the family history before DT administration was an added value for physicians, as it allowed the professional to be prepared for the patient’s emotional states.

#### Distinguishing Dignity Therapy From Advance Care Planning

##### Barriers

The borderline differences between DT and similar types of intervention were highlighted during the training and while engaging in DT with the patient. This realization appeared to be mainly linked to the role of palliative care physicians who usually investigated patients’ will. One of the tasks of the palliative care physicians is also to assess the values and perspectives of the patients about the assistance, examining their needs and preferences.

##### Enablers

Participants highlighted the concept of DT as a helpful intervention in understanding the patients’ values, providing the basis on which to plan subsequent Advance Care Planning.

## Discussion

Our contribution describes how a range of palliative healthcare professionals was affected by the training, incorporated some aspects into their work, and found gain from it. In particular, we described the application of the DT process by two palliative care physicians and how other trainees were influenced and used what they had learned more simply, i.e., to use some DT questions to open a dialogue related to the life experiences in the care relationship.

The possibility of knowing the DT questions and the evidence derived from DT supported the trainees to consider an intervention that helps explicitly to enhance the patient’s dignity. This intervention was not designed to collect quantitative data but to involve healthcare professionals and patients in an emotionally engaging process to create a Generativity Document.

In agreement with previous studies (Chochinov, 2012; Johnston et al., 2016; Korman et al., 2021), trainees confirmed DT as an opportunity to support patients’ life narrative and as a professionally enriching experience since the communication was dedicated to the patient’s life history.

Moreover, the acquisition of DT skills also revealed the training to be helpful beyond DT itself, facilitating the trainees’ engagement in informal communication with patients. In particular, it allowed shifting from “talking to” the patients to talking “with” the patients, as suggested by others’ contributions (Johnston et al., 2016; Aspiras et al., 2019; Korman et al., 2021).

Several trainees reported introducing some of the DT questions learned in their regular communication with their patients. They attributed an empathic approach to the doctor/nurse–patient relationship. As reported in a study, DT emerged from nurses’ words as a powerful intervention, potentially making the patient’s care “holistic” and, therefore, more suitable in fulfilling their diverse needs (Nunziante et al., 2021).

One study suggested that providing patients with an opportunity to talk about life lessons (not only those taught by the illness) and share them with loved ones appears to be feasible. For example, two DT questions particularly salient to generativity (eight and nine DT questions) seem to elicit responses that embrace the meaning of life, regardless of health condition. They, therefore, support both patients and professionals to open a dialogue that is not solely centered on suffering (Buonaccorso et al., 2021).

Professionals in palliative care face many challenging situations and intense feelings that can affect them, i.e., frequently caring for patients with poor prognosis (Emanuel and Librach, 2007). Trainees in our study needed time to learn how to “handle” DT. In particular, many felt they would have needed to implement their psychological skills to administer DT confidently. In another study, the nurses reported that their emotional involvement with the patient made them feel overwhelmed and uncomfortable (Nunziante et al., 2021). They perceived themselves as lacking the expertise needed to deal with advanced cancer patients and with the broad scope of the DT questions, especially concerning coping with the patients’ emotional reactions.

DT offered to the patients the opportunity to address aspects of life they felt most important, such as recounting parts of their life they feel proudest of, things they thought most meaningful, the personal history they would most want to remember, or advice to their family and friends (Chochinov, 2012). In our experience, the trainees suggested that having DT-trained psychotherapists on the team to supervise them as they administer DT would be an opportunity to implement this intervention, as indicated by another study (Nunziante et al., 2021). One of the roles of the psychologist in palliative care is also to support healthcare professionals in implementing psychological skills, i.e., through specific training or the discussion of clinical cases.

Furthermore, supervision and staff support are valuable tools for improving performance and expressing emotions and feelings that can arise within the group or some individuals (Moghaddam, 2014; Adams et al., 2020; Buonaccorso et al., 2022). Nonetheless, this study suggests that healthcare professionals should be trained on the use of DT, as reported in the literature (Montross et al., 2011; Mai et al., 2018; Schoppee et al., 2022).

A recent study examined intervention fidelity among therapists trained with a systematized training protocol (Schoppee et al., 2022). The data showed that fidelity to DT delivery was acceptable for most transcripts and provided insights for improving the consistency of intervention delivery. The authors suggested that the systematized training protocol and ongoing monitoring with the fidelity audit tool will facilitate consistent intervention delivery and add to the literature about fidelity monitoring for brief protocol psychotherapeutic interventions (Schoppee et al., 2022).

Despite the DT protocol not being related to the patients’ awareness of death, the depth of questions might elicit strong emotions (either positive or negative); palliative care physicians chose to administer DT only to patients who were aware of their disease stage. Unlike most other symptom-focused interventions, the beneficial effects of DT reside in being able to bolster a sense of meaning and purpose while reinforcing a continued sense of worth within a supportive, nurturing, and accessible framework, even for those proximate to death (Chochinov, 2012). For example, a study on patients with COPD demonstrated that DT supported coping with death (Brozek et al., 2019). However, in our research, DT was used with cancer patients in the early palliative stage instead of the terminal phase. Therefore, this data have not been thoroughly evaluated.

From an operational point of view, despite DT being distinct from Advance Care Planning, the possibility of collecting data on a patient’s life history and wishes can support palliative care physicians for future Advance Care Planning. If Advance Care Planning becomes a routine activity for physicians, DT may capitalize on the timing devoted to the planning, as reported in recent studies (Dose et al., 2018; Korman et al., 2021).

Finally, several comments from participants concerned the time requested to carry out the protocol (recording, transcription, and editing). They reported that the medical trainer in charge or the volunteers would be essential in optimizing the time for the interview transcription. This aspect in our experience depended on local resources available.

To the best of our knowledge, there are no DT services in Italy. Depending on the resources of the context, it is also possible to take advantage of services dedicated to transcribing. We suggest using automatic transcription devices when it is impossible to have dedicated transcripts. However, since the patient will sign the Generativity Document, we also recommend careful control of the automatic transcription.

Other studies reported that it might not be feasible for busy clinicians to find enough time in their schedules to offer DT to patients, especially with late notice (Weru et al., 2020; Korman et al., 2021). That being said, long-term psychotherapy would often require more clinical hours allocated to each patient. The authors suggested that if future large-scale work demonstrates a low attrition rate for the DT, and similar positive feedback regarding both acceptability and impact, building the capacity to offer DT on a larger scale could be a worthwhile endeavor (Korman et al., 2021).

### Educational Point of View

The training course topics gave rise to a wealth of comments and reflections beyond the two-afternoon training modules. The consideration reported by the participants suggested the importance of offering subsequent briefing for reflection and comparison of the use of DT, even after, and not only during, the training. The fact that only two of 16 participants adhered to the tutored DT session with the patient highlights several aspects. On the one hand, the need for a different training format that allows the participants to become more comfortable with the intervention and for them to fulfill the educational objective of the course.

Taken together and based on our participants’ feedback, we suggest that the training on DT:

(i) Includes more training modules to leave enough time for trainees to explore topics

(ii) Allows extensive discussion among participants

(iii) Foresees a longer time frame for participants to identify the patients with which they would like to attempt DT

(iv) Includes 1-year follow-up meetings on their experience with DT use to share insights or suggestions with colleagues.

These considerations deriving from our experience are confirmed by a recent contribution describing detailed training methods for DT therapists (Schoppee et al., 2021). The DT experts’ verbal and written feedback on the practice and actual sessions encouraged the trainees to provide additional attention to eight components:

(1) Initial framing (i.e., clarifying and organizing the patient’s own goals for creating the legacy document)

(2) Verifying the patient’s understanding of DT

(3) Gathering the patient’s biographical information

(4) Using probing questions

(5) Exploring the patient’s story thread

(6) Refocusing toward the Generativity Document creation

(7) Inviting the patient’s expression of meaningful messages

(8) General DT processes.

In the initial in-person intensive training described in the article, various teaching methods were used. The authors suggested lecture, discussion, demonstration, multimedia presentation, role-playing, and work with case examples.

In our training, role-playing and multimedia presentation were not proposed. Still, examples of DT derived from the clinical experience of the teacher and individual work of reflection on how to use DT in clinical practice were used. As reported by the recent contribution of Schoppee et al. (2021), actively involving participants in the conduction of DT through role-playing and video are indispensable teaching tools. The authors reported that it is vital that all training be led by an expert in the intervention to quickly assess when trainees’ understanding deviates from the original or when intervention consistency is failing.

For future training, trainers supervising the use of DT or research projects should be DT experts. Some articles do not describe the educational path of those who have applied to the DT. This gap also makes it difficult to compare the data and evaluate the application.

In our contribution, the supervision process was offered to two palliative care physicians until the delivery of the Generative Document to the patient. As suggested in the recent description of the training protocol, a training manual was developed to provide a detailed step-by-step guide to implement the DT intervention. The detailed instructions covered each step in the procedure, from the initial screening of the potential patient to the delivery of the final GD.

Offering a training manual to the participants could improve the supervision process carried out by the expert and support the use of the DT in the time following the training. In our study, training material was not available.

Finally, a DT Contact and Processing Tracking Form was proposed by the authors as a guide for the therapist-trainers (Schoppee et al., 2021). In our experience, considering that in Italy, psychotherapy is an intervention that psychotherapists can only be performed, it can be beneficial to organize different trainings for psychotherapists and other healthcare professionals (doctors, nurses, social workers, and chaplains). In particular, DT for non-psychotherapist professional could be proposed as a narrative intervention.

### Limitations and Implications for Future Work

This study describes the experience from a single center and might not entirely represent other oncological centers or other disease settings. Moreover, supervised administration of DT was carried forth and completed by only two palliative care physicians, thus limiting the number of interviews and feedback to be analyzed.

Future studies investigating DT’s use by palliative care physicians in the hospital on a larger sample of patients are desirable. In addition, this is a case study; the effectiveness of the training course was not assessed. Finally, our experience is before the contribution to fidelity of the use of DT (Schoppee et al., 2022) and the training protocol (Schoppee et al., 2021), so our data must be read in the light of these recent contributions.

## Data Availability Statement

The original contributions presented in this study are included in the article/supplementary material, further inquiries can be directed to the corresponding author.

## Ethics Statement

Ethical review and approval was not required for the study on human participants in accordance with the local legislation and institutional requirements. Written informed consent for participation was not required for this study in accordance with the national legislation and the institutional requirements.

## Author Contributions

LB: conceptualization. LG and AA: methodology. LB and LG: writing and original draft preparation. LB and ST: review and editing. SA: review. All authors have read and agreed to the published version of the manuscript.

## Conflict of Interest

The authors declare that the research was conducted in the absence of any commercial or financial relationships that could be construed as a potential conflict of interest.

## Publisher’s Note

All claims expressed in this article are solely those of the authors and do not necessarily represent those of their affiliated organizations, or those of the publisher, the editors and the reviewers. Any product that may be evaluated in this article, or claim that may be made by its manufacturer, is not guaranteed or endorsed by the publisher.

## Footnotes

1 www.eapcnet.eu/Corporate/AbouttheEAPC/Definitionandaims/tabid/151/Default.aspx (last accessed 25/07/2011).

2 https://www.who.int/news-room/fact-sheets/detail/palliative-care (last accessed 05.08.2020).
